# Genome Sequence of the Self-Flocculating Strain Saccharomyces cerevisiae SPSC01

**DOI:** 10.1128/genomeA.00367-18

**Published:** 2018-05-17

**Authors:** Jian-Ren Xu, Lei-Yu He, Chen-Guang Liu, Xin-Qing Zhao, Feng-Wu Bai

**Affiliations:** a School of Life Science and Biotechnology, Dalian University of Technology, Dalian, China; b School of Life Science and Biotechnology, Shanghai Jiao Tong University, Shanghai, China

## Abstract

The self-flocculation of yeast cells presents advantages for continuous ethanol fermentation such as their self-immobilization within fermenters for high density to improve ethanol productivity and cost-effective biomass recovery by gravity sedimentation. We sequenced and analyzed the genome of the self-flocculating Saccharomyces cerevisiae SPSC01 for the industrial production of fuel ethanol.

## GENOME ANNOUNCEMENT

High productivity cannot be achieved for regular Saccharomyces cerevisiae isolates, since unicellular yeast cells cannot be retained within fermenters for high density under continuous fermentation conditions (1). When yeast cells self-flocculate, not only can they be retained within fermenters for high density to improve ethanol productivity (2), but they are more tolerant to ethanol for high product titers (3). The self-flocculating S. cerevisiae SPSC01 has been commercialized in fuel ethanol production (4).

SPSC01 was sequenced by the whole-genome shotgun approach (5). Paired-end short reads were generated with about 793 Mb of raw data and 577 Mb of clean data. The reads were assembled and validated using SOAPdenovo (http://soap.genomics.org.cn/soapdenovo.html) and SOAP aligner version 2.2 (http://soap.genomics.org.cn/soapaligner.html). GenomeScan (http://genes.mit.edu/genomescan.html) and Augustus (http://bioinf.uni-greifswald.de/webaugustus/prediction/create) were used to predict the open reading frames (ORFs), which were annotated by their best match according to BLAST analysis (https://blast.ncbi.nlm.nih.gov/Blast.cgi) and aligned using CLUSTALW (http://www.genome.jp/tools/clustalw). ORFs larger than 100 bp were included as candidates. For filtered ORFs, gene annotation was based primarily on comparisons to the Saccharomyces Genome Database (http://www.yeastgenome.org), the Saccharomyces Species Database (http://www.broadinstitute.org/annotation/fungi/comp_yeasts), as well as the Clusters of Orthologous Groups (COG)/KEGG (6, 7), Swiss-Prot (8), TrEMBL (9), and nonredundant (NR) (10) databases. Noncoding RNAs, including rRNAs and tRNAs, were predicted by RNAmmer (http://www.cbs.dtu.dk/services/RNAmmer) and tRNAScan (http://lowelab.ucsc.edu/tRNAscan-SE). Repeated sequences were identified via aligning the assembly with the repetitive sequence database using RepeatMasker (http://www.repeatmasker.org/cgi-bin/WEBRepeatMasker) and Repeat Protein Masker (11). The tandem repeat sequences were analyzed using Tandem Repeat Finder (12). Single nucleotide polymorphisms (SNPs), insertion-deletions, and copy number variations were analyzed by using the Short Oligonucleotide Analysis Package (SOAPsnp) (http://soap.genomics.org.cn/index.html). Phylogenetic analyses were carried out using CLUSTALW. Prediction and annotation of RNA genes were manually performed based on the results of BLASTn searches of the SPSC01 genome with the S288c sequences of these genes and elements as queries. Comparison of genome chromosome synteny between the model S. cerevisiae S288c (13) strain and the Brazilian industrial isolate S. cerevisiae JAY291 (14) was performed using the MUMmer software (15).

The SPSC01 genome is ∼15 Mb, encompassing a capacity for 5,315 genes with an average length of 1,532 bp. The genome coverage depth of 33.59 resulted in a final assembly of 11,372,406 bp with a GC content of 38.24%. The complete assembly includes 304 scaffolds, with a scaffold *N*_50_ size of ∼115 kb. Analysis of the SPSC01 scaffolds indicated 1.54% repetitive content. Compared to S288c, SPSC01 has 80,388 SNPs, 2,983 insertions, and 2,991 deletions. Different mutation types and positions of SNPs in the coding sequence, intergenic regions, and introns were classified quantitatively, through which 50,165 SNPs (62.4%) were identified in the coding regions and 30,223 SNPs (37.6%) in the noncoding regions. A phylogenetic tree of SPSC01 was constructed in comparison with published genome sequences of yeast strains, which demonstrated that SPSC01 is close to S. cerevisiae Kyokai no. 7, which is used for fermentation to produce sake in Japan (16). Many differences were observed in the genome of SPSC01 by synteny analysis compared to that of S288c and the industrial strain JAY291.

### Accession number(s).

This whole-genome shotgun sequence has been deposited at DDBJ/EMBL/GenBank under the accession no. NPJN00000000. The version described in this paper is the first version, NPJN01000000.

## References

[1] Bai FW, Anderson WA, Moo-Young M 2008 Ethanol fermentation technologies from sugar and starch feedstocks. Biotechnol Adv 26:89–105. doi:10.1016/j.biotechadv.2007.09.002. 17964107

[2] Zhao XQ, Bai FW 2009 Yeast flocculation: new story in fuel ethanol production. Biotechnol Adv 27:849–856. doi:10.1016/j.biotechadv.2009.06.006. 19577627

[3] Zhao XQ, Bai FW 2009 Mechanisms of yeast stress tolerance and its manipulation for efficient fuel ethanol production. J Biotechnol 144:23–30. doi:10.1016/j.jbiotec.2009.05.001. 19446584

[4] Xu TJ, Zhao XQ, Bai FW 2005 Continuous ethanol production using self-flocculating yeast in a cascade of fermentors. Enzyme Microb Technol 37:634–640. doi:10.1016/j.enzmictec.2005.04.005.

[5] Metzker ML 2010 Sequencing technologies—the next generation. Nat Rev Genet 11:31–46. doi:10.1038/nrg2626. 19997069

[6] Kanehisa M, Araki M, Goto S, Hattori M, Hirakawa M, Itoh M, Katayama T, Kawashima S, Okuda S, Tokimatsu T, Yamanishi Y 2008 KEGG for linking genomes to life and the environment. Nucleic Acids Res 36:D480–D484. doi:10.1093/nar/gkm882. 18077471 PMC2238879

[7] Tatusov RL, Fedorova ND, Jackson JD, Jacobs AR, Kiryutin B, Koonin EV, Krylov DM, Mazumder R, Mekhedov SL, Nikolskaya AN, Rao BS, Smirnov S, Sverdlov AV, Vasudevan S, Wolf YI, Yin JJ, Natale DA 2003 The COG database: an updated version includes eukaryotes. BMC Bioinformatics 4:41. doi:10.1186/1471-2105-4-41. 12969510 PMC222959

[8] Bairoch A, Boeckmann B, Ferro S, Gasteiger E 2004 Swiss-prot: juggling between evolution and stability. Brief Bioinform 5:39–55. doi:10.1093/bib/5.1.39. 15153305

[9] Boeckmann B, Bairoch A, Apweiler R, Blatter MC, Estreicher A, Gasteiger E, Martin MJ, Michoud K, O'Donovan C, Phan I, Pilbout S, Schneider M 2003 The SWISS-PROT protein knowledgebase and its supplement TrEMBL in 2003. Nucleic Acids Res 31:365–370. doi:10.1093/nar/gkg095. 12520024 PMC165542

[10] Huson DH, Auch AF, Qi J, Schuster SC 2007 MEGAN analysis of metagenomic data. Genome Res 17:377–386. doi:10.1101/gr.5969107. 17255551 PMC1800929

[11] Korf I 2004 Gene finding in novel genomes. BMC Bioinformatics 5:59. doi:10.1186/1471-2105-5-59. 15144565 PMC421630

[12] Benson G 1999 Tandem repeats finder: a program to analyze DNA sequences. Nucleic Acids Res 27:573–580. doi:10.1093/nar/27.2.573. 9862982 PMC148217

[13] Goffeau A, Barrell BG, Bussey H, Davis RW, Dujon B, Feldmann H, Galibert F, Hoheisel JD, Jacq C, Johnston M, Louis EJ, Mewes HW, Murakami Y, Philippsen P, Tettelin H, Oliver SG 1996 Life with 6000 genes. Science 274:546, 563–567. doi:10.1126/science.274.5287.546. 8849441

[14] Argueso JL, Carazzolle MF, Mieczkowski PA, Duarte FM, Netto OVC, Missawa SK, Galzerani F, Costa GGL, Vidal RO, Noronha MF, Dominska M, Andrietta MGS, Andrietta SR, Cunha AF, Gomes LH, Tavares FCA, Alcarde AR, Dietrich FS, McCusker JH, Petes TD, Pereira GAG 2009 Genome structure of a *Saccharomyces cerevisiae* strain widely used in bioethanol production. Genome Res 19:2258–2270. doi:10.1101/gr.091777.109. 19812109 PMC2792172

[15] Kurtz S, Phillippy A, Delcher AL, Smoot M, Shumway M, Antonescu C, Salzberg SL 2004 Versatile and open software for comparing large genomes. Genome Biol 5:R12. doi:10.1186/gb-2004-5-2-r12. 14759262 PMC395750

[16] Akao T, Yashiro I, Hosoyama A, Kitagaki H, Horikawa H, Watanabe D, Akada R, Ando Y, Harashima S, Inoue T, Inoue Y, Kajiwara S, Kitamoto K, Kitamoto N, Kobayashi O, Kuhara S, Masubuchi T, Mizoguchi H, Nakao Y, Nakazato A, Namise M, Oba T, Ogata T, Ohta A, Sato M, Shibasaki S, Takatsume Y, Tanimoto S, Tsuboi H, Nishimura A, Yoda K, Ishikawa T, Iwashita K, Fujita N, Shimoi H 2011 Whole-genome sequencing of sake yeast *Saccharomyces cerevisiae* Kyokai no.7. DNA Res 18:423–434. doi:10.1093/dnares/dsr029. 21900213 PMC3223075

